# Management of microbial keratitis by private pharmacies in Uganda: a study of knowledge, attitude, and practice.

**DOI:** 10.12688/wellcomeopenres.22594.1

**Published:** 2025-01-27

**Authors:** Abel Ebong, Simon Arunga, Tara B. Mtuy, Angela Meric Birungi, John Onyango, Gilbert Arinda, Francis Orishaba, Teddy Kwaga, Rita Kageni, David Macleod, Reena Yadav, Sandip Das Sanyam, Jeremy J. Hoffman, Abeer H. A. Mohamed Ahmed, Astrid Leck, Mathew J. Burton

**Affiliations:** 1 Department of Ophthalmology, Mbarara University of Science and Technology, Mbarara, Western Region, Uganda; 2 London School of Hygiene & Tropical Medicine, International Centre for Eye Health, London, UK; 3 Kilimanjaro Christian Medical College, Moshi, Tanzania; 4 Sagarmatha Choudhary Eye Hospital, Lahan, Nepal; 5 National Institute for Health Research Biomedical Research Centre for Ophthalmology at Moorfields Eye Hospital NHS Foundation Trust and UCL Institute of Ophthalmology, London, UK

**Keywords:** Microbial Keratitis, Bacterial keratitis, Fungal keratitis, Keratitis, Traditional Eye Medicine, Pharmacies, Uganda

## Abstract

**Purpose:**

To determine the knowledge, attitude, and practice of pharmacy attendants in the management of microbial keratitis.

**Methods:**

This mixed-methods study was conducted in selected pharmacies and drug shops located in Mbarara City between March and May 2022. We administered questionnaires assessing the knowledge, attitudes, and practices (KAP) related to microbial keratitis (MK) to 140 pharmacy attendants (PAs) in the drug shops and pharmacies. We also conducted 40 in-depth interviews (IDI) and three focus group discussions (FGD) with pharmacy attendants to discuss practices, challenges, and opportunities to improve the management of MK.

**Results:**

Of the 140 pharmacy attendants, almost half (49.29%) reported that they were not confident when making diagnoses in patients with eye problems, and 19.29% were uncertain of which drug to prescribe. In the IDIs and FGDs, the pharmacy attendants reported that they receive and manage patients with MK. Some immediately refer any patients they receive with eye complaints without prescribing any medication while others only refer those who are not responding to medication. The challenges faced in managing patients with MK included inadequate knowledge of managing eye diseases including MK, patients presenting with severe diseases because of delays in seeking healthcare, and the use of traditional eye medicine. The pharmacy attendants suggested ways of improving the management of MK and other eye diseases in the community including community sensitisation on eye diseases and conducting continued professional development lecture sessions on MK in pharmacies, drug shops, and clinics.

**Conclusion:**

The study showed that probable MK was a common presentation among patients seeking to buy drugs from pharmacies and drug shops. Pharmacies are key stakeholders in the health seeking journey of patients and hence need to be supported through capacity building and strengthening the referral network to improve the outcomes of patients with eye diseases and MK.

## PLAIN LANGUAGE SUMMARY

Microorganisms like bacteria, fungi, viruses or protozoa can cause corneal infections. Corneal infections are a common and serious eye problem that leads to loss of sight and high morbidity for affected individuals in many parts of Africa. Corneal infections is a time-sensitive disease and once people present late, little can be done to reverse the damage and mitigate a poor outcome

Over the past few years, our group has been conducting formative research in Uganda to understand the epidemiology, presentation journeys and management challenges to support interventional packages to prevent needless blindness from MK. A major challenge has been delayed presentation to the appropriate level health facility where patients can receive effective care.

A mapping of the patient journey of patients with corneal infections showed that those who first visited local pharmacies were 45% and 47% in Uganda and Nepal respectively. In this context, local pharmacy shops play an important role as the primary point of consultation. However, there is a gap in our understanding of the knowledge of these key stakeholders on corneal infections and their management of patients with eye diseases including corneal infections presenting at their drug shops and pharmacies.

We conducted this study as part of our intervention development process to better understand the knowledge and practice of corneal infection management by the private pharmacies in Uganda. This was to inform the implementation of an early screening and referral interventional cluster randomised trial in Uganda as a prevention strategy for severe corneal infections.

## Introduction

Microbial keratitis (MK) refers to infection of the cornea by causative organisms like bacteria, fungi, viruses, or protozoa. Globally, it is estimated that each year more than a million people develop fungal keratitis and several times that develop bacterial keratitis ^1^. It is a common and serious eye problem that leads to loss of sight and high morbidity for affected individuals in many parts of Africa. In southwestern Uganda the majority of patients with MK present at hospitals with severe disease and about 9% of the affected eyes are eviscerated ^2^.

Over the past few years, our group has been conducting formative research in Uganda to understand the epidemiology, presentation journeys and management challenges to support interventional packages to prevent needless blindness from MK. A major challenge has been delayed presentation to the appropriate level health facility where patients can receive effective care ^3^. MK is a time-sensitive disease and once people present late, little can be done to reverse the damage and mitigate a poor outcome ^4^. There are various studies that indicate the importance of interventions at the primary level setting to decrease the severity of microbial keratitis ^5– 8^.

Our current work shows that delay was mediated, among other things, through poor triage, and referral in the health system, as well as the use of traditional eye medicine (TEM) ^9^. The proportion of MK patients that visited local pharmacies was 45% and 47% in Uganda and Nepal respectively ^3, 10^. In this context, local pharmacy shops play important role as the primary point of consultation. However, there is a gap in our understanding of the knowledge and practice for treatment of MK among these key stakeholders.

We conducted this study as part of our intervention development process to better understand the knowledge and practice of MK management by the private pharmacies in Uganda. This was to inform the implementation of an early screening and referral interventional cluster randomised trial in Uganda as a prevention strategy for severe MK.

## Methods

### Study settings and participants

The study was conducted in Mbarara district, South-Western Uganda. Mbarara is the regional capital of 17 districts with a population of 5,000,000 people. There are three tertiary eye referral hospitals located in Mbarara. Study data collection was conducted from 7 ^th^ June 2022 to 26 ^th^ July 2022.

Our study population was pharmacy attendants working at pharmacies and drug shops located within Mbarara district. We used a mixed methods descriptive cross-sectional study design with a Knowledge, Attitudes, and Practices (KAP) questionnaire, in-depth interviews (IDIs), and focus group discussions (FGDs) to collect quantitative and qualitative data.

Based on our previous work, only 12% of the health workers in the primary health system could make a correct diagnosis of MK, a sample size of 124 out of the 507 registered pharmacies and drug shops in Mbarara would be sufficient estimate the proportion to within a margin of error of 5% using a 95% CI. To allow for an approximate non-response rate of 10%, 140 pharmacies and drug shops were selected ^11^.

In Uganda, drug shops are health care shops that sell over-the-counter drugs; these over-the-counter drugs are classified as class C drugs. Drug shops are registered and managed by nurses, nursing assistants and pharmacy assistants. They are permitted to sell a restricted list of medicines. On the other hand, pharmacies are larger health care shops that in addition to selling Class C drugs, they can also sell class A and class B. Class A and class B Group I drugs are supplied under prescription by a registered medical practitioner, dentist, or veterinary person. Class B group II drugs may be supplied by retail only by a registered pharmacist or licensed pharmacy. Therefore, pharmacies in Uganda are registered and supervised by pharmacists ^12^. However, the day to day running, such as dispensing of over the counter and prescription medicine is done by registered nurses, enrolled nurses or pharmacy assistants.

In Uganda, enrolled nurses and midwives are health workers who have a certificate in nursing and midwifery respectively, while registered nurses and midwives are health workers with diplomas in nursing and midwifery.

### KAP survey

Trained nurse interviewers collected data using KAP questionnaires, from 140 pharmacy attendants working in pharmacies and drug shops located in Mbarara district. The pharmacies and drug shops were selected using a computer randomising function in Microsoft excel. In each selected pharmacy or drug shop, one pharmacy attendant on duty at the time was interviewed. The KAP questionnaire can be found in the supplementary material as supplementary material 1 (Extended data). Briefly, this questionnaire had 15 questions designed to assess knowledge, attitudes towards MK diagnosis and management and practice patterns in identifying, managing and referral of MK. The questionnaire had a mix of multiple-choice answer questions and open-ended structured questions. It was administered by trained research assistants (nurses) who ticked the correct options or wrote down free text answers based on how the pharmacy attendants responded to the asked questions.

### In-Depth Interviews (IDIs)

The pharmacies and drug shops that had participated in the KAP survey were called and informed of the in-depth interviews we intended to conduct; we then went ahead to interview those that accepted to participate. The pharmacy attendants were called until we reached the required sample size of 40 participants. We purposely selected based on cadres of pharmacy attendants to ensure representation of the different cadres working in pharmacies and drug shops. The IDIs were conducted with the guidance of an IDI topic guide by the same research assistants who conducted the KAP survey. The IDI topic guide can be found in the supplementary material as supplementary material 2 (Extended data). The discussions were audio recorded on a phone. The IDI topic guide included questions on the ability of the pharmacy assistants to recognise patients with MK, and how often they see patients with MK. We explored how the pharmacy attendants manage these patients and challenges they face in managing patients with MK. We also discussed circumstances under which pharmacy attendants would refer these patients to the eye hospitals and any challenges they face when referring patients with MK to the eye hospitals. The pharmacy attendants were also asked to give suggestions on ways of improving the management of MK.

### Focus Group Discussions

The participants of the FGDs were selected from pharmacies and drug shops that had participated in both the KAP quantitative survey and the IDIs. The selection was based on their responses in the IDIs, having reported seeing patients with signs of microbial keratitis at their pharmacies and drug shops. The discussions, facilitated by the principal investigator and the study coordinator, were guided by a topic guide. The topics guide we used in the focused group discussion can be found in the supplementary material as supplementary material 3 (Extended data). The discussions were audio recorded on a phone. During the FDGs we discussed participants’ experiences in managing patients with MK and other eye diseases, ways to improve care and referral mechanisms.

### Bias

Since this was a predominantly interview based study. The reliability of the responses would have been largely subjective. To minimise recall bias, additionally qualitative methods (IDIs and FDGs) were used to triangulate the individual responses.

### Data management and analysis

Quantitative data were entered into a Microsoft Access database and imported into STATA 16 for analysis (Although these are licensed platforms, the data can also be entered and analysed fully in Microsoft excel). Only participant identification numbers were entered in the Microsoft Access alongside the data. The data was stored in a password protected computer only accessible to the study team. The questionnaires filled with participant responses were kept in a secure locker.

Simple tabulations were used to summarise descriptive data on the knowledge and practice of the pharmacy attendants on MK. The answers for each question were independently analysed as proportions of the responses.

Qualitative data was manually processed. All interviews were conducted in English. The audio records of the IDIs and FGDs were transcribed. Summaries from transcripts and field notes were generated for analysis. Abel Ebong (AE) manually coded the data guided by the research question, KAP questionnaire and topic guides with emphasis on the challenges that pharmacy attendants faced in the management of patients with MK. The coding was verified by Simon Arunga (SA). Using thematic analysis, emerging themes identified from coded data were discussed and verified with the study team. Data were synthesised and presented in a narrative text by themes.

This manuscript was prepared following the Strengthening the Reporting of Observational Studies in Epidemiology (STROBE) and Standards for Reporting Qualitative Research (SRQR) guidelines. The completed STROBE and SRQR checklist can be accessed at reporting guidelines.

## Results

### Demographic characteristics of the study respondents

In the KAP survey, we interviewed 140 pharmacy attendants working in different pharmacies and drug shops in Mbarara. Most of the pharmacy attendants interviewed were enrolled nurses 100 (71%). The median age of the participants was 26 years (IQR 25 – 28, total range 21 – 40 years). Most pharmacies and drug shops (104, 74%) were located in semi-urban areas in the district. In the IDIs, we interviewed 23 (58%) enrolled nurses, 4 (10%) pharmacists, 2 (5%) pharmacy technicians and 11 (28%) registered nurses who worked in 40 different pharmacies and drug shops. The median age of participants in the IDI was 27 years (IQR 26 – 28 years, total range 23 – 35 years). Twenty-four pharmacy attendants participated in the focus group discussions. These included 14 enrolled nurses (58%), 6 registered nurses (25%), 2 pharmacists (8%), and 2 pharmacy technicians (8%). Their median age was 27 years (IQR 25 – 28, total range 23 – 25 years). A summary of the sociodemographic characteristics of the study participants who participated in the study is represented in Table 1 below.

In the results section, we present results of the KAP, IDIs and FGDs arranged into four themes: knowledge of MK, challenges of managing MK, challenges to referring patients, and solutions.

**Table 1.** Table showing the sociodemographic characteristics of the pharmacy attendants who participated in the KAP survey.

Variable	n/140	%
**Sex**		
Female	88	63
Male	52	37
**Professional training of the pharmacy attendant**		
Pharmacist	6	4
Pharmacy Technician	5	3
Registered nurse	29	21
Enrolled Nurse	100	71
**Type of facility**		
Pharmacy	45	32
Drug shop	95	68
**Location of pharmacy/drug shop**		
Urban ^a^	36	26
Semi-urban ^b^	104	74

^a^Urban areas are areas within the central business areas of the district ^b^Semi-urban areas are areas within Mbarara City but outside the central business area.

### Knowledge on Microbial Keratitis

The responses of the participants have been summarised in Table 2. The knowledge of pharmacy attendants was variable for different eye conditions, but generally low. In the IDIs, few of the participants could identify the signs and symptoms of microbial keratitis. Some of the participants interviewed reported that they had no knowledge of how patients with microbial keratitis present; while others reported that they had never heard of microbial keratitis.

**Table 2.** Knowledge and practice of managing MK (microbial keratitis) in our sample of pharmacy attendants.

Variable	n/140	%
**How often do you receive patients with eye related problems?**		
About 1 – 2 per day	22	17
> 2 per day	10	7
About 1 – 2 per week	38	27
About 1 – 2 per in a fortnight	11	8
< 1 – 2 per month	15	11
About 1 – 2 per month	23	16
Can’t say exactly	21	15
**What do you think are the causes of a painful red eye?**		
Conjunctivitis	97	69
Cataract	5	3
Xerophthalmia	2	1
Corneal abrasion	21	15
Injury	47	14
What challenges do you face when dealing with customers seeking advice for eye related problems? ^*^		
Diagnosing the disease	69	49
Knowing which eye drop to give	28	20
Varies from patient to patient	58	41
Don’t know if a drug is working in a patient	21	15
Knowing when to refer	18	13
Don’t know	1	1
In what situations can topical steroids be correctly used in the eye? ^*^		
Patient with a red eye	68	48
Patient with painful eye	57	41
Topical steroids are only appropriate for selected cases	39	28
Steroids can delay wound healing and suppress the body’s immunity	25	18
Steroids can raise the pressure in the eye	13	9
I am unsure whether to use them in the eye	13	9
**How would you advise a patient with a red eye related to minor trauma? (n=139)**		
Prescribe antibiotics	19	14
Prescribe steroids	5	4
Prescribe steroids in combination with antibiotic steroid eye drops/ointment	32	23
Prescribe oral medication (tablets)	3	2
Directly refer to an eye hospital without giving anything	28	20
Prescribe antibiotics and refer them to an eye hospital urgently	20	14
Prescribe combination therapy (antibiotics and steroids) and refer urgently	32	23
What do you know about corneal infections? ^*^		
Is a whitish appearance in the black part of the eye	57	41
Is very serious, if not promptly treated a person can lose their sight	60	43
Minor abrasion following trauma may result in corneal infection if not treated promptly	42	30
It usually gets better without treatment	1	1
**Do you think corneal blindness is an important issue in your community**		
Yes	74	53
No	40	29
Don’t know	25	18
**What initiatives can be implemented in partnership with pharmacy owners to reduce corneal blindness resulting from eye infections**		
Emphasizing the need to refer all cases to a health facility or an eye Centre/hospital	37	27
Instruct which antibiotics to prescribe before urgently referring cases to a specialist eye hospital	22	16
Providing training for pharmacists to enable them to recognize and treat minor eye conditions	78	57
No answer	1	1

* Questions in which more than one response was permitted hence had percentages that are more than 100%

*“I have never heard of it (corneal ulcers).”* [ID1-3, nurse]
“ *I know a few of the eye conditions, but specifically I don’t know anything about corneal ulcers*”. [IDI-4, nurse]

As a result of this limited knowledge, in the KAP survey, 49% of the participants reported challenges diagnosing patients with eye diseases. The limited knowledge about microbial keratitis was attributed to the training the respondents had received while in school. A nurse (IDI-1) reported that *“we do little things in schools, so you find we don’t have enough knowledge on that condition (microbial keratitis).”* Another nurse (FGD–04) reported that.

“ *There are eye conditions, though you have been to school, you have never seen it. You just read it in a book and have never come across it. Even with your 10 years of experience, you have never come across it.”*

This inadequate knowledge they reported affected their ability to manage patients and their confidence when treating patients with eye diseases.

“…. *so, this client comes and most of the things we are not sure. Is it a bacterial infection or is it allergic? So, you find yourself giving a combined drug. So, if it is bacterial, you are treating it. That is why you find we miss out on fungal infections”.* [FGD -02, nurse]

### Challenges in management of MK

In addition to poor knowledge, several challenges impacting the management of MK were reported by participants. In the KAP, many pharmacy attendants (49%) were uncertain about making an MK diagnosis, 20% did not know which treatment to give and 13% did not know when to refer.

Pharmacy attendants in the IDIs and FGDs reported self-prescribing to be a challenge. A significant proportion of patients were reported to present to the pharmacy to buy medicine rather than first seek care from a health facility. They also discussed some patients who refuse drugs prescribed by doctors citing no improvement and the lack of availability of some drugs prescribed.

“ *Patients come asking for a specific medicine most of the time. When you try to ask them for the prescription, you find they are going to treat themselves for either trauma, allergy, eye infection, and that is a big percentage of the clients we get in the pharmacies”.* [FGD-03, nurse]
*“Sometimes when we are dispensing a drug which was recently prescribed to the patient but that didn’t help them, someone says; ‘you are giving me this one, I used it, and it didn’t help me in any way.’* [FGD-5, a pharmacist]
“S *ometimes a doctor prescribes a very rare drug, and the patient sometimes spends almost 3 weeks looking for that drug”.* [FGD-4, pharmacist]

The cost of medicine was also noted to be a major challenge. The pharmacy attendants reported financial considerations for the patients could lead to a less effective treatment for the patients. In some circumstances where the doctor’s prescription is expensive or not available, patients request the pharmacy attendants to prescribe alternative drugs.

*“Sometimes the patient tells you, I don’t have the money to buy what the doctor has written. So can I get something for the money I have”*. [FGD-8, nurse]

Participants noted the strong belief and frequent use of traditional eye medicines as a significant challenge leading to severe disease and posing an additional challenge in managing the condition. The use of traditional eye medicine was believed to be mostly about beliefs in its effectiveness as a treatment regardless of one’s economic status in some cases.

“ *There is a problem that patients do not want to seek medical care, even if some have money. They prefer to use traditional medicine*.” [FGD-9, a nurse]

The diagnostic capacity of pharmacy attendants was discussed as a challenge. In instances of uncertainty, they refer the patients to eye hospitals where they can be examined well. A nurse reported.

*“Some patients come to the pharmacies and say; ‘I have a thing here in the eye, but if I try to examine the eye, I don’t even see the thing.”* [IDI-1]
“ *We know we don’t have eye examination machines and we know we don’t have eye specialists here, so we refer”.* [IDI-3, a nurse]

### Challenges in referral of patients with MK

In the KAP survey, most of the pharmacy attendants (58%) reported that they would refer a patient with a red eye to an eye hospital. When they were asked about the pre-referral management they would give to the patient; 20% responded that they would prefer to urgently refer directly to an eye hospital without giving any medicines; while 14% responded that they would prefer to urgently refer after they have given an antibiotic, and 23% responded that they would prefer to urgently refer but after they have given a combination of antibiotic and steroid. 

We explored the challenges the pharmacy attendants faced when referring patients with MK to an eye hospital. The reported challenges included inadequate information about the referral eye hospitals, weak coordination between the primary facilities and referral centres, patients’ attitudes, and cost.

Most participants did not know what services are offered in some tertiary eye hospitals and the charges they levy. They reported this affected their ability to effectively counsel the patients being referred to the hospitals.

“ *Sometimes patients request information about the referral sites, for example information about the hospital fees, but we find we cannot give them adequate information about the hospitals we are referring them to, because we also don’t have adequate information about the hospitals”.* [FGD-12, a nurse]

The pharmacy attendants reported that they do not have telephone contacts of eye specialists and the eye hospitals and hence are unable to consult them and inform them when they are referring to them patients.

*“Calling the eye hospital directly for example on a hotline would be the most appropriate way of managing an eye emergency and referring patients. But since we don't have the contacts of the eye hospitals, we can only verbally tell them to the eye clinic at the general referral without information the hospital.”* [FGD-15, a nurse]

It was reported that some patients do not want to be referred to government eye hospitals because they are doubtful that they will receive the level of care required to manage their eye condition. They noted that long waiting times, poor customer care, health workers’ absenteeism and lack of drugs in the hospitals affect patient acceptance of referrals. It was reported that patients trusted the service of drug shops.

“ *Patients are biased that whenever they go to the government eye hospitals, they find a line of patients waiting to be attended to. For instance, you may wait for 3 hours to be attended to. So, they tell you, I am not going to get the help I need where you are sending me. So, if you are not going to attend to me, let me go to another outlet [pharmacy] where I will get what I want”.* [FGD-6, nurse]
“ *Our patients believe that in private clinics and private drug shops we handle them well because we want money*.” [FGD-2, nurse]

### Proposed solutions to challenges in managing MK

We explored potential solutions to support the pharmacy attendants in managing and referring patients with probable MK. In the KAP, about 56% of the respondents proposed training of pharmacists to enable them to recognise and treat minor eye conditions, 30% proposed a strengthened referral to the eye hospitals, while 15% proposed guidance on initial first line treatment and referral.

One solution was to sensitise pharmacies on how to care for patients with MK. They discussed a training of Pharmacy attendants that would include management of MK and consistent messaging to the patients. This information would then filter out to the community.

“ *…. I think the first community is us who work in the clinics, pharmacies, and the drug shops. For example, what you have done today (conducting the FGDs) is very excellent. But I wish you to extend this (conducting the FGD) to all people working in drugs shops and pharmacies. So that when someone comes to my pharmacy, and I refer, and then goes to another pharmacy and they also refer and are getting the same message, it will make the patients more confident that we are giving them the right information”.* [FGD-03, a nurse]

In addition, pharmacy attendants suggested trainings include guidelines to help improve their diagnosis of patients with eye diseases. Some pharmacy attendants suggested training be given in the form of continuous medial education (CME) sessions.

“ *For us on the maternity ward, you find guidelines’ chart so that for a given disease we know we can do this and this. You can also provide printouts and give to us in pharmacies so that even those who don’t attend the trainings or CME sessions can also benefit*”. [FGD-02, nurse]

To support referrals to the eye hospitals, the pharmacy attendants suggested improved communication channels to enable teleconsultations and allow for facilitated referrals.

*“By availing contacts of the specialists, when we get them [patients with eye diseases], we can call you and tell you the history so that the patient feels comfortable before coming. Then we write something small. We can write their name and put on that paper the phone contact of the person we are referring to”.* [FGD-11, pharmacist]
*“If we had a hotline directly to the eye clinic, it would be better because that would be the most appropriate way of managing an emergency. But we don't have the contacts of the eye hospitals; hence we only verbally tell them to the eye clinic at the general referral.”* [FGD-15, a nurse]

## Discussion

This study among pharmacies in Uganda is part of a programme of work seeking to understand the dynamics of management of Microbial Keratitis (MK), a major cause of corneal blindness in Uganda. In this study, we found that pharmacies play a significant role in the management of eye patients, some of whom could potentially have MK. However, despite this “leading role”, there were major self-identified challenges faced by the pharmacy attendants in diagnosis, management and referral of patients suspected with MK.

As noted in our previous studies, most eye patients in Uganda present first at the local pharmacies and drug shops for treatment ^10^. In this study, more than half of the pharmacy attendants reported that they see at least one patient with an eye disease per week; a large majority of whom present without prescriptions. Unlike in most countries, the pharmacy regulations in Uganda “allow” over the counter dispensing of a vast range of medicines apart narcotics and controlled drugs. Consequently, many patients prefer buying medicines from the nearest drug shops and pharmacies and only go to a clinic or hospital when there is no improvement ^11^.

As anticipated, knowledge about microbial keratitis among pharmacy attendants was low. In this study most pharmacy attendants reported challenges in correctly diagnosing eye diseases. This was also noted in our previous work when we assessed the health system and the knowledge among primary health workers who included enrolled nurses, clinical officers, medical officers, and ophthalmic clinical officers ^11^. This limited knowledge on management of eye diseases can exacerbate the disease, leading to worse outcomes. We note an important opportunity, however, that there is willingness among the pharmacy attendants to engage in training and receive support with guidelines on management of eye diseases.

In addition to the low levels of knowledge, we noted that the pharmacies and drug shops lacked basic diagnostic tools to aid in the identification of patients with probable MK. We had a similar finding in our previous work on primary health facilities in Uganda ^11^. Inexpensive tools like a torch and fluorescein stain strips can help primary health workers to identify patients with a corneal epithelial defect ^11^. We recently published a guide on how to make low-cost fluorescein strips in resource limited settings ^13^. Ideally this requires a torch that has a blue light filter. Recently, a portable, low cost, direct ophthalmoscope (ArcLight) has proved to be a useful tool that can provide blue light visualisation of the cornea and a macro lens for a magnified examination ^14^. Our group is currently exploring how to utilize these tools to help in the early identification of MK in the primary health setting in Uganda and Nepal.

Regarding the referral patterns, we found that most pharmacy attendants preferred to provide treatment, sometimes along with a referral or refer later either with a written referral note or verbally if no improvement was noted. This was seemingly associated with financial incentives to sell their medicine. The potential danger with this is that more often than not, this initial medicine could be ineffective and/or potentially dangerous in cases of MK. For example, we found that none of the pharmacies or drug shops had a stock of antifungal eye medicine. Yet, our previous work showed that about 62% of the patients presenting with MK had fungal keratitis in this region of Uganda ^2^. Even among the pharmacy attendants who were willing to refer, they reported challenges of lack of information about the potential referral hospitals such as hospital contact, working hours, fee structures, waiting times and screening processes. This undermined their capacity to appropriately support the patients in the referral. This is an area that can be strengthened by providing the pharmacy attendants with contact information for the referral hospital and through numerous activities that encourage interactions between the pharmacy attendants and the eye hospital, like conducting continuing medical education sessions. We have previously described the process of facilitated referral of patients with probable MK from the primary health facilities to the regional eye hospitals ^3^.

This study provided further understanding on the challenges of diagnosis, management, and referral of patients with probable MK. This was a key gap that had not been previously covered in earlier studies of the primary health system in Uganda. This mixed methods approach was preferred to enable triangulation of our findings. This information contributes to our ongoing work to improve the identification and management of MK in the primary health setting.

## Conclusion

This study among pharmacy attendants on the management of MK noted that probable MK was a common presentation. However, challenges in diagnosis, management, and referral were identified. Pharmacies are key stakeholders in the health seeking journey of patients and hence need to be supported through capacity building to improve the outcomes of patients with eye diseases, including MK.

## Ethics and consent statement

The study adhered to the Declaration of Helsinki. It was approved by the London School of Hygiene & Tropical Medicine, UK Ethics Committee (ref: 22123) on 20 ^th^ July 2020, Mbarara University Research Ethics Committee (ref: MUREC 1/7) on 1 ^st^ September 2020, and Uganda National Council for Science and Technology (ref: HS1082ES) on 25 ^th^ May 2021. All the study respondents provided Written informed consent prior to data collection including consent for interviews and discussions to be audio recorded.

## Data Availability

Data and documentation that support the study findings are available in the LSHTM Data Compass repository at https://doi.org/10.17037/DATA.00004231 ^15^ as KAP_Pharmacy_dataset. The KAP Pharmacy data was collected with an ethical commitment to participants that it would be stored confidentially and securely, which prevents open access. The ethical body that approved this study was the Mbarara University of Science and Technology Research Ethics Committee (ref: MUREC 1/7). The condition for the approval was to have a regulated data sharing plan with collaborators. Therefore, researchers wishing to access the data are invited to complete a request form in the Data Compass repository, outlining the data variables in which they are interested. Once the dataset has been prepared, the applicant will be asked to agree to a short license agreement and provided with a copy of the data without undue restrictions. The interview and focus group data sets have not been made publicly available due to the identifiable nature of the data. The data sets are available on request by writing to the corresponding author (AE). LSHTM Data Compass: Uganda Pharmacy attendants KAP Survey dataset, https://doi.org/10.17037/DATA.00004231 ^15^. *This project contains the following **underlying** data:* **KAP_supplementary_material:** This file contains the KAP questionnaire that was used to collect the quantitative data; and the topic guides that were used in the in-depth interviews and focus group discussions. This is available as open access. **Participant information sheet:** This is the consent form the participants were required to sign before participating in the KAP survey, in-depth interviews and focus group interviews. **Data request form:** This is the form any research who wants access to the KAP survey dataset shall be required to complete. LSHTM Data Compass: STROBE and SRQR checklist for ‘Management of microbial keratitis by private pharmacies in Uganda: a study of knowledge, attitude, and practice’. https://doi.org/10.17037/DATA.00004499 ^16^. Data are available under the terms of the Creative Commons Zero "No rights reserved" data waiver (CC0 1.0 Public domain dedication).

## Submission statement

This submission has not been published anywhere previously and it is not simultaneously being considered for any other publication.
