# Supplementary material for: Management of microbial keratitis by private pharmacies in Uganda: a study of knowledge, attitude, and practice
Source: Wellcome Open Res. 2025 Jan 27;10:30. [Version 1] doi: 10.12688/wellcomeopenres.22594.1 (PMC12378391; doi:10.12688/wellcomeopenres.22594.1)
Supplement: Peer review history

# Reviewer report: Reviewer response for version 1

**DOI:** 10.21956/wellcomeopenres.24892.r126864

**Published:** 2025-08-25

**Authors:** Jennifer Enright

**Affiliations:** 1 Washington University in St Louis, St. Louis, Missouri, USA

The authors conducted this study to better understand barriers to treatment of microbial keratitis in urban and suburban Uganda. They focused on the role of pharmacy and drug store staff by conducting surveys, interviews and focus group discussions to explore background knowledge, treatment paradigms and referral patterns for MK. A power analysis was conducted to determine the scope of study, which seems appropriate with staff at 124 of 507 pharmacies and drug shops included. Their data highlight areas that can be targeted with interventions including improving the knowledge base of staff regarding diagnosis and treatment of MK, and streamlining the referral process.

 By working directly with pharmacy and drug store staff the authors were able to successfully identify action items that should help improve treatment of MK and visual outcomes for patients. It would be interesting in a future publication to repeat this study after interventions have been implemented. Some of the barriers to care that were identified may be specific to this population- which will be very helpful in designing appropriate interventions but may limit direct application in other communities. However, this study could be used as a model for obtaining similar information in other settings.

Is the work clearly and accurately presented and does it cite the current literature?

Yes

If applicable, is the statistical analysis and its interpretation appropriate?

Not applicable

Are all the source data underlying the results available to ensure full reproducibility?

Yes

Is the study design appropriate and is the work technically sound?

Yes

Are the conclusions drawn adequately supported by the results?

Yes

Are sufficient details of methods and analysis provided to allow replication by others?

Yes

Reviewer Expertise:

Clinical ophthalmology

I confirm that I have read this submission and believe that I have an appropriate level of expertise to confirm that it is of an acceptable scientific standard.

# Reviewer report: Reviewer response for version 1

**DOI:** 10.21956/wellcomeopenres.24892.r127140

**Published:** 2025-08-19

**Authors:** Travis K Redd

**Affiliations:** 1 Casey Eye Institute, Portland, USA

This is an excellent mixed methods study evaluating the current comfort level and performance of pharmacies in Uganda for managing microbial keratitis, as well as methods to improve this performance. I have only a few minor comments:

 1. It is unclear what antimicrobial therapy (if any) can be prescribed by pharmacies in Uganda. Please specify this.

 2. How were the KAP questions validated? For example, “what do you think are the causes of a painful red eye?” appears to have multiple correct answers (corneal abrasion, injury, conjunctivitis), why were respondents forced to select only one answer?

Is the work clearly and accurately presented and does it cite the current literature?

Yes

If applicable, is the statistical analysis and its interpretation appropriate?

Yes

Are all the source data underlying the results available to ensure full reproducibility?

Yes

Is the study design appropriate and is the work technically sound?

Yes

Are the conclusions drawn adequately supported by the results?

Yes

Are sufficient details of methods and analysis provided to allow replication by others?

Yes

Reviewer Expertise:

microbial keratitis, population health research

I confirm that I have read this submission and believe that I have an appropriate level of expertise to confirm that it is of an acceptable scientific standard.
